# Fractal features of soil particle size distribution under different land-use patterns in the alluvial fans of collapsing gullies in the hilly granitic region of southern China

**DOI:** 10.1371/journal.pone.0173555

**Published:** 2017-03-16

**Authors:** Yusong Deng, Chongfa Cai, Dong Xia, Shuwen Ding, Jiazhou Chen

**Affiliations:** 1 Key Laboratory of Arable Land Conservation (Middle and Lower Reaches of Yangtze River) of the Ministry of Agriculture, College of Resources and Environment, Huazhong Agricultural University, Wuhan, People’s Republic of China; 2 College of hydraulic and Environmental engineering, China Three Gorges University, Yichang, China; Peking University, CHINA

## Abstract

Collapsing gullies are among the most severe soil erosion problems in the tropical and subtropical areas of southern China. However, few studies have examined the relationship of soil particle size distribution (PSD) changes with land-use patterns in the alluvial fans of collapsing gullies. Recently, the fractal method has been applied to estimate soil structure and has proven to be an effective tool in analyzing soil properties and their relationships with other eco-environmental factors. In this study, the soil fractal dimension (D), physico-chemical properties and their relationship with different land-use patterns in alluvial fans were investigated in an experiment that involved seven collapsing gully areas in seven counties of southern China. Our results demonstrated that different land-use patterns of alluvial fans had a significant effect on soil physico-chemical properties. Compared to grasslands and woodlands, farmlands and orchards generally contained more fine soil particles (silt and clay) and fewer coarse particles, whereas significant differences were found in the fractal dimension of soil PSD in different land-use patterns. Specifically, the soil fractal dimension was lower in grasslands and higher in orchards relative to that of other land-use patterns. The average soil fractal dimension of grasslands had a value that was 0.08 lower than that of orchards. Bulk density was lower but porosity was higher in farmlands and orchards. Saturated moisture content was lower in woodlands and grasslands, but saturated hydraulic conductivity was higher in all four land-use patterns. Additionally, the fractal dimension had significant linear relationships with the silt, clay and sand contents and soil properties and exhibited a positive correlation with the clay (R^2^ = 0.976, P<0.001), silt (R^2^ = 0.578, P<0.01), organic carbon (R^2^ = 0.777, P<0.001) and saturated water (R^2^ = 0.639, P<0.01) contents but a negative correlation with gravel content (R^2^ = 0.494, P<0.01), coarse sand content (R^2^ = 0.623, P<0.01) and saturated hydraulic conductivity (R^2^ = 0.788, P<0.001). However, the fractal dimension exhibited no significant correlation with pH, bulk density or total porosity. Furthermore, the second-degree polynomial equation was found to be more adequate for describing the correlations between soil fractal dimension and particle size distribution. The results of this study demonstrate that a fractal dimension analysis of soil particle size distribution is a useful method for the quantitative description of different land-use patterns in the alluvial fans of collapsing gullies in southern China.

## Introduction

Soil is a porous medium composed of soil particles of different sizes and shapes [1], and its structure and properties are determined by soil particle size distribution (PSD), which indirectly affects soil moisture characteristics, fertility and soil erosion [2]. During the last few decades, many soil scientists have used the PSD to predict physical properties such as water retention, bulk density, permeability, and porosity [3–6]. To a certain extent, PSD is an important index for the evaluation of soil and its relationship with other soil functions [7–8]. In areas with high soil erosion rates due to rainfall and runoff, fine particle-size fractions (accompanied by nutrients) are selectively removed or deposited during soil erosion processes [9–10]. A number of previous studies have proposed that land use largely influences PSD by promoting or hindering soil erosion [11–14]. Therefore, the characterization of PSD can help to reveal the influence of land use on soil properties.

A quantitative description of soil PSD is important for soil structure research. During the last few decades, several different methods have been established for determining soil PSD [15–17]. Textural analysis is commonly used to characterize soil PSD, but the size definitions of the three main particle fractions (sand, silt and clay) are rather arbitrary and thus cannot provide complete information about the soil PSD. Additionally, in the textural triangle, soils grouped in a textural class exhibit a wide PSD range (e.g., the silt loam in the textural triangle contains soils that roughly vary between 50% and 80% in silt content), thus providing incomplete information regarding PSD [9]. Fractal theory, which is a method of describing systems with non-characteristic scales and self-similarity, was first proposed and established by Mandelbrot (1977) [18]. In recent years, this theory has been utilized for quantitative descriptions of soil PSD characteristics and has attracted the attention of pedologists worldwide. Tyler and Wheatcraft (1992) [19] developed a mass-based distribution to estimate the fractal dimension of PSD and developed the limits of fractal behavior and applications for soil PSD. Based on the mass-based approach, Yang et al. (1993) [20] applied fractal scaling theory in describing soil PSD characteristics of different soil textures in northern China. The results of their studies demonstrated that fractal dimension analysis was very sensitive to clay content. Later, Bittelli et al. (1999) [21] characterized PSD using a fragmentation model based on the mass fractal dimension in three domains: clay, silt, and sand. Huang and Zhan (2002) [22] found that the fractal dimension of PSD increased with clay content but decreased with sand content. Millan et al. (2003) [23] analyzed the relationship between scaling exponents and soil texture on the basis of the fractal model. Their research demonstrated that the fractal dimension of soil PSD was significantly positively correlated with clay content following a linear trend. Su et al. (2004) [24] demonstrated that fractal dimensions of PSD are useful parameters in monitoring soil degradation and desertification processes. Additionally, Prosperinin and Perugini (2008) [25] systematically described the characteristics of soil particle size distribution by utilizing the fractal model in the Umbria region of Italy. More recently, Segal et al. (2009) [26] and Arya et al. (2010) [27] used the fractal method and theory to identify soil PSD as a useful factor for predicting soil hydraulic properties and compared the results with those determined using traditional methods. Furthermore, many multifractal measures have been used to characterize PSD by analyzing the specific data of soil particles [9, 28–31]. Despite extensive research on soil structure using the fractal theory, to our knowledge, few studies have focused on the influence of land-use patterns on the fractal dimension of soils [32–33].

**Fig 1:**
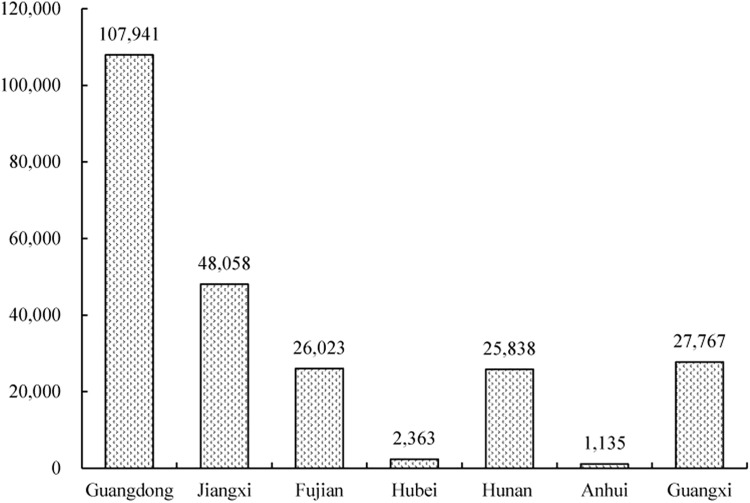
Number of collapsing gullies in areas of southern China. Data are from Feng et al. (2009).

**Fig 2:**
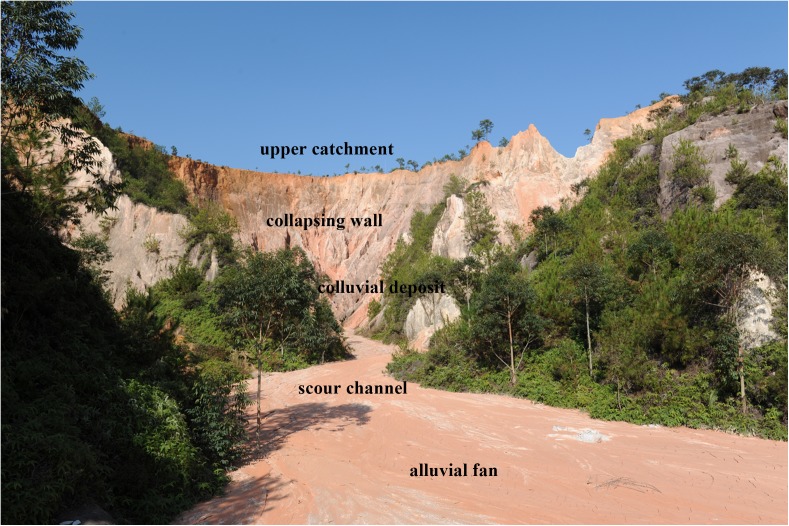
A typical collapsing gully in the hilly granitic region of Anxi County, Fujian Province (photo: Shuwen Ding), consisting of (1) upper catchment; (2) collapsing wall; (3) colluvial deposit; (4) scour channel; (5) alluvial fan.

**Fig 3:**
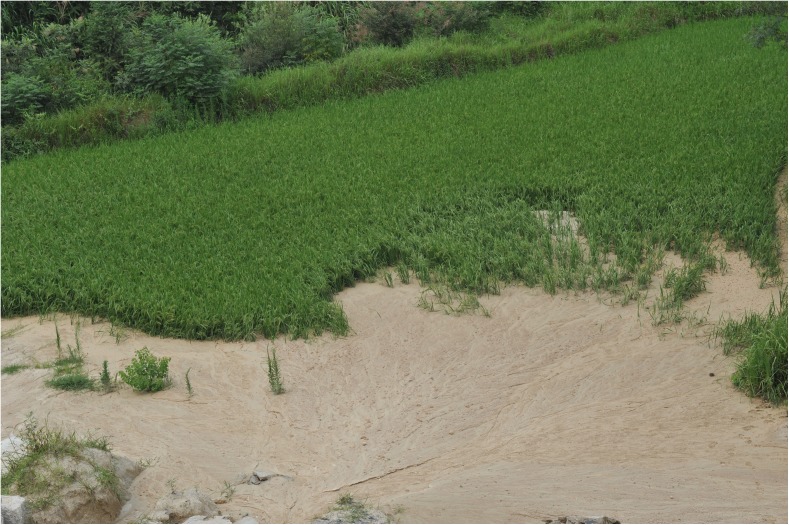
Farmland covered by an alluvial fan from a collapsing gully in Tongcheng County, Hubei Province (photo: Shuwen Ding)

Water loss and soil erosion pose great threats to China’s environment and economy [34]. In particular, gully erosion is an important indication of land degradation, rendering slopes unfit for agriculture and representing an important source of sediment in a range of environments [35]. First described by Zeng (1960) [36], collapsing gully erosion, a widely distributed erosional phenomenon in the hilly granitic region of southern China, is caused by a combination of intense runoff and gravity in areas that receive more precipitation than the soil is able to absorb [37–40]. These gullies develop quickly and collapse suddenly, with an annual average erosion rate of more than 50 kt km^-2^ yr^-1^, which is more than 50 times greater than the erosion rate on gentler slopes or on slopes with high vegetation cover [41]. According to a 2005 survey by the Monitoring Center of Soil and Water Conservation of China, there are hundreds of such degraded ecosystems in the hilly granitic region of southern China, and the number of collapsing gullies is estimated to be 239,100 within an area that extends from 21°01’–32°05’N and 106°49’–120°27’E, including the provinces of Guangdong, Jiangxi, Hubei, Hunan, Fujian, Anhui, and Guangxi (Fig 1) [42–43]. A collapsing gully system consists of five parts: (1) an upper catchment, where a large amount of water is accumulated; (2) a collapsing wall, where mass-soil wasting, water erosion and gravitational erosion are quite severe; (3) a colluvial deposit, where residual material is deposited; (4) a scour channel, where the sediment accumulation and transport is usually deep and narrow; and (5) an alluvial fan, the zone below the gully mouth, where sediments transported by the collapse are deposited (Fig 2) [37, 39, 44]. From 1950 to 2005, collapsing gully erosion affected 1,220 km^2^ in the granitic red clay soil region, leading to the loss of more than 60 Mt of soil [43]. The collapsing gullies in turn led to the losses of 360,000 ha of farmland, 521,000 houses, 36,000 km of road, 10,000 bridges, 9,000 reservoirs, and 73,000 ponds, as well as an economic loss of 3.28 billion USD that affected 9.17 million residents [38, 45]. As part of collapsing gully systems, alluvial fans are sedimentary regions and are the most destructive features to farmlands in collapsing gully areas (Fig 3). When collapsing gully erosion occurs, the cultivation layer of farmlands in alluvial fans is covered by a large amount of sediment, which severely affects the physico-chemical properties of the soil and further diminishes the crop yield. Several researchers have investigated collapsing gully erosion in China, but most studies have focused on the formation mechanism and governance of collapsing gully erosion [46–49]. As a result, few studies have been performed on the effects of land use on the soil quality of these degraded land areas.

In this study, we investigated the PSD and fractal dimensions (D) of soil in seven different alluvial fans of collapsing gullies at different latitudes in the hilly granitic region of southern China. Our objectives were to (i) determine the effect of different land-use patterns on the soil properties and soil PSD of alluvial fans of collapsing gullies in different regions; (ii) describe and assess the status of the fractal dimensions of different land-use patterns in these alluvial fans and investigate the relationship between soil fractal dimensions and soil properties; and (iii) explore the possibility of using the fractal dimension of soil PSD as an integrating index for quantifying soil PSD characteristics and soil degradation extent of these alluvial fans. This study lays a theoretical foundation for further research regarding soil protection, soil recovery mechanisms, and other soil science topics concerning alluvial fans in collapsing gullies.

## Materials and methods

### Study area

This study was conducted in seven counties of southern China, including Tongcheng County (TC) in Hubei Province, Longhui County (LH) in Hunan Province, Gan County (GX) in Jiangxi Province, Changting County (CT) and Anxi County (AX) in Fujian Province, Wuhua County (WH) in Guangdong Province and Cangwu County (CW) in Guangxi Province. These counties were selected as the study sites because they suffer from the most severe collapsing gully erosion in the hilly granitic region of southern China. The field study does not involve endangered or protected species and these sites do not require specific permission.

TC (29°02’–29°24’N, 113°36’–114°04’E), located in southeastern Hubei Province in central China, has a temperate monsoon continental climate, an average temperature of 15.5–16.7°C, average annual precipitation of approximately 1,550 mm with high variability, and an accumulated temperature (> 10°C) of 5,058°C. The region is dominated by granitic red soil, with granites in the area formed during the Yanshanian period. Many granitic joints formed by the Yanshan movement and inherited from the bedrock facilitate slope failure. According to a 2005 survey by the Yangtze River Water Resources Commission of the Ministry of China, TC has 1,102 collapsing gullies that contribute to an annual soil loss of more than 120 million ha, accounting for 58.4% of the total soil erosion loss in the county [50].

LH (27°00’–27°40’N, 110°15’–110°38’E), located in southwestern Hunan Province, is characterized by a subtropical monsoon climate, an annual average temperature of 11–17°C, annual precipitation of 1,427.5 mm, a rainy season from April to September that accounts for 76% of the total annual precipitation, and an annual average frost-free period of 281.2 days. The region is dominated by granitic red soil, with granites in the area formed during the Caledonian period, and there are 1,617 collapsing gullies in the county [51].

GX (25°26’–26°17’N, 114°42’–115°22’E), located in southern Jiangxi Province, has a characteristically warm subtropical humid monsoon climate, a mean annual temperature of 19.3°C, average annual sunshine of 1,092 h, average annual rainfall of 1,394.3 mm, an average of 160 rainy days per year, and a frost-free period of 298 days. The region is dominated by granitic red soil, with the granites in the area formed during the early Caledonian and Yanshanian periods. Soil erosion is severe in this area, and there are 4,138 collapsing gullies in the county [52].

CT (25°18’–26°02’N, 116°00’–116°39’E), located in western Fujian Province, belongs to the subtropical monsoon climate zone; the climate is warm, the mean annual temperature is 18.3°C, the average annual rainfall is 1,685.6 mm, and the frost-free period is 260 days. The dominant soil type is red soil, and the granites in the area were formed during the Yanshanian period. CT has 1,592 collapsing gullies [53].

AX (24°50’–25°26’N, 117°35’–118°17’E), located in southeastern Fujian Province, is characterized by a typical subtropical maritime monsoon climate, an annual average sunshine of 2,069 h, an annual average temperature of 16–22°C, and an accumulated temperature (>10°C) of 5,000–7,700°C. The average annual precipitation is 1,600 mm, with 81% occurring between March and September and an average of 160 rainy days per year. The average frost-free period is 350 days. The dominant soil type is red soil, and the granites in the area were formed during the Yanshanian period. AX has 4,744 collapsing gullies, the most of all counties in Fujian Province [54].

WH (23°23’–24°12’N, 115°18’–116°02’E), located in northeastern Guangdong Province, is characterized by a subtropical monsoon climate, an annual average temperature of 20°C, and annual precipitation of 1,547.5 mm, and the rainy season is concentrated between April and September, accounting for 76% of the total annual precipitation. The granites in the area were formed during the Yanshanian period. There are a total of 22,117 collapsing gullies in WH, representing a serious threat to the local economy [55].

CW (22°58’–24°10’N, 110°51’–111°40’E), located in the eastern part of the Guangxi Zhuang autonomous region, is characterized by a subtropical monsoon climate, an average annual temperature of 21.12°C and average precipitation of 1,500 mm. CW has 1,592 collapsing gullies, 580 of which are in the town of Longxu [56].

### Soil sampling

Extensive alluvial fans form at the mouth of most gullies in the study area. Most valleys are covered by alluvial fans, and essentially all previous farmlands have been abandoned. However, some alluvial fans are used as farmland areas by local farmers due to a shortage of agricultural land. This study was conducted to investigate the expansion of seven collapsing gully areas in southern China, including TC, LH, GX, CT, AX, WH and CW. These regions are all experiencing an expansion in collapsing gullies, resulting in the accumulation of alluvial fan sediments. Additionally, four land-use patterns, including farmland, orchard, woodland and grassland, were chosen as areas of focus among the alluvial fans of the collapsing gullies in the study area. All soil types in the study area are granitic red soils (Ultisols). Soil samples were collected from the four different land-use patterns at a total of 28 sampling sites (i.e., 7 locations × 4 land uses). At each of these sites, we removed the litter and selected 10–20 points in the tillage layer in an S-pattern under different land-use patterns. Then, the soil collected from several points was combined into a single sample, and approximately 1–2 kg of soil samples was transferred to the laboratory after a four-way division of all samples. Additionally, bulk density, porosity and soil moisture were determined by using metal cylinders whose volumes were approximately 100 cm^3^ (5.02 cm diameter and 5.05 cm height).

### Soil analysis

Each soil sample was dried at laboratory room temperature (25°C) to a constant weight and then sieved through a 2-mm mesh to remove roots, stone and other debris for experimental analysis. According to the United States Department of Agriculture classification of soil particle size, soil PSD was described in terms of the percentages of gravel (1–2 mm), coarse sand (0.5–1 mm and 0.25–0.5 mm), fine sand (0.1–0.25 mm and 0.05–0.1 mm), silt (0.002–0.05 mm) and clay (<0.002 mm). Sand fractions were obtained using the wet sieving method, and silt and clay fractions were determined using the pipette method after the removal of organic matter using hydrogen peroxide and heat treatment with sodium hexametaphosphate as the dispersing agent. Each PSD sample was measured in triplicate to minimize the underestimation of primary silt and clay particles and to achieve good reproducibility.

The soil physical and chemical properties were tested according to the procedures described by the Institute of Soil Science of the Chinese Academy of Sciences (ISSCAS, 1978) [57]. Bulk density (BD) was measured by collecting samples from the 0–20 cm soil layer using metal cylinders whose volumes were approximately 100 cm^3^ (5.02 cm diameter and 5.05 cm height), followed by weighing the samples and then drying (105°C) them to a constant weight. Soil porosity was calculated using bulk density (BD) and particle density (PD; 2.65 mg m^-3^) according to the following equation: total porosity (%) = (1-BD/PD)×100. Saturated water content was measured using the gravimetric method, saturated hydraulic conductivity was measured according to the constant head method, soil pH was determined with the potentiometric method on a soil/water suspension (1:2.5 ratio), and soil organic matter (SOM) was quantified with the K_2_Cr_2_O_7_-H_2_SO_4_ oxidation method of Walkey and Black [58].

### Measurement of soil fractal dimension

The fractal dimension can be defined by the relationship between number and size in a statistically self-similar system (Mandelbort 1982) [59]:

$$N(X>x_{i})=kx_{i}{}^{\text{-}D}$$ (1)

where *N(X>x*_*i*_*)* is the cumulative number of objects or fragments greater than a characteristic size *x*_*i*_, *k* is the number of elements at a unit length scale, and *D* is the fractal dimension. The applicability of Eq (1) for PSD analysis, however, is limited due to the incomplete and inaccurate calculations of N from conventional experimental data. To solve this problem, Tyler and Wheatcraft [19] determined D using Eq (2):

$$\frac{M(r<R_{i})}{M_{T}}={\left(\frac{R_{i}}{R_{\max}}\right)}^{3\text{-}D}$$ (2)

where *M* (*r<R*_*i*_) is the cumulative mass of soil particles of the *i*^th^ size with *r* less than *Ri*, *M*_*T*_ is the total mass (g) of the soil lower than *R*_*max*_, *R*_*i*_ is the soil particle radius, *R*_*max*_ is the maximum particle radius, and *D* is the soil particle fractal dimension. Eq (2) can be rearranged in a log-log scale, providing a method for determining the fractal dimension from the slope of the regression log [*M(r<R)/M*_*T*_] versus log [*R*_*i*_ */R*_*max*_]. The slope of the graph of log [*M(r<R)/M*_*T*_] versus log [*R*_*i*_ */R*_*max*_] is (3-*D*). Eq (2) was used for analysis in this study.

### Statistical analysis

The data were analyzed using one-way analysis of variance (ANOVA) with the SPSS 19.0 software package. The least significant difference (LSD) procedure was used to separate the means of these soil properties at a significance level of P<0.05, and all of the results are reported as the mean±SD (standard deviation). Regression analysis was used to analyze the relationship of the fractal dimension (*D*) with the content of soil particles and soil physico-chemical properties.

## Results

### Soil particle-size distributions

As shown in Table 1, there were considerable differences in soil PSD under the four land-use patterns in the seven alluvial fans of collapsing gullies. The predominant soil particle sizes were found to be silt (0.002–0.05 mm) and coarse sand (0.25–1.0 mm), followed by gravel (1.0–2.0 mm), fine sand (0.05–0.25 mm) and clay (<0.002 mm). The percentages of silt ranged between 18.77% in grasslands and 28.62% in woodlands of TC, 28.14% in grasslands and 36.59% in woodlands of LH, 20.46% in farmlands and 28.35% in grasslands of GX, 16.72% in woodlands and 32.85% in farmlands of CT, 17.66% in farmlands and 37.61% in orchards of AX, 15.83% in woodlands and 30.13% in orchards of WH, and 25.36% in woodlands and 28.58% in grasslands of CW. The concentration of coarse sand and gravel for the soils of the four land-use patterns showed significant group characteristics, and the coarse sand concentration was higher than that of gravel in TC, GX and AX. The fine sand concentration was greater in farmland than in the other land-use patterns in LH, AX and WH. The highest concentrations of clay in TC, LH, GX, CT, AX, WH and CW were 16.54% in orchards, 19.99% in orchards, 17.29% in orchards, 19.56% in farmlands, 18.06% in orchards, 17.98% in orchards and 20.22% in farmlands, respectively. We can conclude from the texture classification that the soil texture varies under the four land-use patterns in the seven alluvial fans. The soil texture is sandy loam for farmlands, woodlands and grasslands in TC; grasslands in LH; and farmlands in GX. It is sandy clay loam for the orchards in TC; farmlands in LH; orchards, woodlands and grasslands in GX; woodlands and grasslands in CT; farmlands, woodlands and grasslands in AX; farmlands in WH; and all the land-use patterns in CW. The soil texture is clay loam for the orchards and woodlands in LH, farmlands and orchards in CT, orchards in AX, and orchards in WH.

**Table 1:** Different particle-size distributions under different land-use patterns in the alluvial fans of collapsing gullies (%).

Code		Soil particle-size distribution (mm)									Soil texture
		Gravel	Coarse sand		Total of coarse sand	Fine sand		Total of fine sand	Silt	Clay	
		2.0–1.0	1.0–0.5	0.5–0.25		0.25–0.1	0.1–0.05		0.05–0.002	<0.002	
TC	Farmland	18.02±0.20b	14.16±0.14a	8.64±0.31c	22.80	11.39±0.73a	11.64±1.37b	23.03	23.02±0.69c	13.13±0.55b	Sandy loam
	Orchard	19.35±0.11ab	13.21±0.24ab	9.32±0.22c	22.53	7.34±0.30b	7.79±1.18c	15.12	26.46±1.34b	16.54± 0.46a	Sandy clay loam
	Woodland	19.22±0.11ab	11.04±0.67c	13.52±0.42a	24.56	5.79±0.23c	10.97±0.77b	16.76	28.62±2.18a	10.84±1.51c	Sandy loam
	Grassland	20.11±0.19a	14.41±0.90a	11.72±0.58b	26.12	10.39±0.60a	14.95±3.51a	25.34	18.77±2.32d	9.67±0.69c	Sandy loam
LH	Farmland	17.57±0.52c	9.55±0.38c	9.41±1.35a	18.97	9.82±0.19b	8.95±1.24b	18.77	29.60±1.57b	15.09±0.97b	Sandy clay loam
	Orchard	19.24±0.76b	10.71±0.85b	5.29±0.23b	16.01	4.82±0.34c	9.72±1.55a	14.54	30.22±1.39b	19.99±1.20a	Clay loam
	Woodland	15.47±0.37d	10.05±0.91b	9.89±1.28a	19.94	3.68±0.81c	8.86±0.70b	12.54	36.59±2.29a	15.47±0.76b	Clay loam
	Grassland	21.69±1.76a	12.61±0.57a	6.93±0.36b	19.55	11.38±0.55a	7.32±0.34c	18.71	28.14±1.91c	11.92±1.31c	Sandy loam
GX	Farmland	18.49±0.68ab	14.44±1.22b	8.14±0.33b	22.58	12.49±1.22a	16.46±1.64a	28.95	20.46±1.08c	9.53±0.50bc	Sandy loam
	Orchard	10.56±0.48b	13.94±0.53b	10.46±0.55a	24.40	8.30±0.42c	13.62±0.89b	21.92	25.83±2.01b	17.29±2.51a	Sandy clay loam
	Woodland	21.37±0.64a	17.63±0.79a	4.44±0.39c	22.07	10.80±0.56b	11.07±0.76c	21.87	25.70±3.12b	8.99±1.57c	Sandy clay loam
	Grassland	20.21±0.66a	13.02±0.87b	10.05±0.24a	23.07	10.14±0.76b	7.69±1.00d	17.83	28.35±1.69a	10.54±1.20b	Sandy clay loam
CT	Farmland	12.76±1.54d	8.48±0.63c	8.13±0.30b	16.61	7.58±0.50c	10.64±3.19b	18.22	32.85±1.95a	19.56±0.62a	Clay loam
	Orchard	18.70±1.05c	10.06±1.17b	5.93±0.29c	16.00	7.12±0.47c	13.02±0.65a	20.14	30.06±3.62ab	15.10±0.50b	Clay loam
	Woodland	20.57±0.64b	16.61±1.15a	11.16±0.96a	27.78	10.52±0.73b	14.59±1.85a	25.12	16.72±0.32c	9.81±1.24d	Sandy clay loam
	Grassland	23.23±2.23a	15.87±1.90a	5.94±0.27c	21.80	14.19±0.04a	9.11±1.07b	23.30	20.47±2.13b	11.20±0.63c	Sandy clay loam
AX	Farmland	24.29±1.11a	15.45±0.86b	12.84±0.75b	28.29	10.45±0.59b	11.17±1.87a	21.62	17.66±0.31c	8.14±1.92c	Sandy clay loam
	Orchard	11.03±0.97c	8.35±0.39d	6.41±0.28d	14.76	12.99±0.05a	5.54±0.38c	18.54	37.61±0.65a	18.06±0.68a	Clay loam
	Woodland	21.58±1.64b	12.30±0.90c	9.35±0.28c	21.65	10.35±0.47b	10.57±0.20a	20.93	22.55±1.21b	13.29±0.37b	Sandy clay loam
	Grassland	19.85±0.25bc	17.66±0.40a	14.44±0.16a	32.10	9.31±0.01c	9.21±0.59b	18.52	20.83±1.49bc	8.70±0.41c	Sandy clay loam
WH	Farmland	17.70±0.77c	12.56±0.43c	8.76±0.21b	21.32	10.74±1.27a	10.02±0.59a	20.76	27.65±1.15ab	12.57±0.86b	Sandy clay loam
	Orchard	14.74±0.71d	14.69±0.49b	7.27±0.28c	21.96	3.50±0.22c	11.70±3.00a	15.20	30.13±2.05a	17.98±1.85a	Clay loam
	Woodland	26.25±2.86b	21.24±1.05a	11.63±1.23a	32.87	9.54±0.44b	6.01±1.87b	15.55	15.83±0.86b	9.49±1.74c	Loamy sand
	Grassland	30.03±0.33a	21.18±1.13a	8.37±0.35b	29.54	9.02±0.29b	6.90±0.89b	15.92	16.37±2.48b	8.14±0.19c	Loamy sand
CW	Farmland	17.38±0.54bc	11.28±0.33a	4.71±0.28c	15.99	6.24±0.21b	12.09±0.26a	18.32	28.09±1.23a	20.22±0.68a	Sandy clay loam
	Orchard	14.33±0.69c	9.08±0.79b	10.161.12b	19.24	10.43±0.23a	9.92±0.67b	20.35	26.69±0.25b	19.38±1.92a	Sandy clay loam
	Woodland	25.24±1.45a	8.37±0.69b	14.39±0.33a	22.76	5.24±0.22b	11.34±2.23ab	16.58	25.36±0.44b	10.06±1.74c	Sandy clay loam
	Grassland	19.67±0.63b	12.70±0.26a	8.38±0.43b	21.08	3.85±0.36c	12.26±0.72a	16.11	28.58±2.07a	14.55±0.44b	Sandy clay loam

### Soil fractal dimension

According to the double-logarithmic model of the soil PSD fractal dimension from Eq (2), $\mathrm{lg}\left(\frac{M(r<R_{i})}{M_{T}}\right)$ and $\mathrm{lg}\left(\frac{R_{i}}{R_{\max}}\right)$ were linearly fitted utilizing the least-squares method to obtain the soil mass fractal dimension values of the land-use patterns in the alluvial fans of collapsing gullies in TC, LH, GX, CT, AX, WH and CW. The relevant parameters of calculation are shown in Table 2. The highest fractal dimension values for the land-use patterns in each of the alluvial fans were orchards in TC (2.748), orchards in LH (2.779), orchards in GX (2.745), farmlands in CT (2.769), orchards in AX (2.759), orchards in WH (2.705) and farmlands in CW (2.777). The soil particle fractal dimension was higher when the soil texture was finer, corresponding to the soil PSD. The results demonstrated that the fractal dimensions of woodlands and grasslands are smaller than those of the other land-use patterns in most cases, and a lower fractal dimension leads to a higher concentration of both gravel (1.0–2.0 mm) and coarse sand (0.25–1.0 mm), indicating that the soil texture is coarser in the land-use patterns of the alluvial fans. As shown in Table 2, soil D was determined to range between 2.631 and 2.829 in TC, 2.571 and 2.822 in GX, 2.697 and 2.871 in AX, 2.579 and 2.810 in WH, and 2.611 and 2.846 in CW. The range in fractal dimension values was 2.661–2.748 with an average of 2.701 in TC, 2.703–2.779 with an average of 2.739 in LH, 2.658–2.745 with an average of 2.687 in GX, 2.662–2.769 with an average of 2.714 in CT, 2.639–2.759 with an average of 2.690 in AX, 2.649–2.760 with an average of 2.695 in WH, and 2.679–2.777 with an average of 2.738 in CW. The data for each of the alluvial fans revealed a strong linear trend and strong correlations as indicated by the two R^2^ values greater than 0.90 (Table 2). Overall, despite the relatively narrow soil D values in this study, we conclude that certain land-use patterns can improve soil D values, especially farmlands and orchards. Thus, when compared with PSD or soil properties, soil D values may provide additional information regarding the restoration of land subjected to desertification in the alluvial fans of collapsing gullies. This possibility will be further discussed in a later section.

**Table 2:** Soil fractal dimension of different land-use patterns in the alluvial fans of collapsing gullies.

Soil sample	TC		LH		GX		CT		AX		WH		CW	
	D	R^2^	D	R^2^	D	R^2^	D	R^2^	D	R^2^	D	R^2^	D	R^2^
Farmland	2.708	0.994	2.733	0.979	2.658	0.985	2.769	0.964	2.639	0.998	2.705	0.982	2.777	0.976
orchard	2.748	0.990	2.779	0.969	2.745	0.988	2.736	0.965	2.757	0.950	2.76	0.971	2.764	0.991
woodland	2.687	0.974	2.741	0.945	2.661	0.978	2.662	0.994	2.713	0.995	2.666	0.991	2.679	0.981
grassland	2.661	0.993	2.703	0.981	2.683	0.98	2.688	0.997	2.649	0.997	2.649	0.992	2.732	0.972

### General soil characteristics of alluvial fans

**Fig 4:**
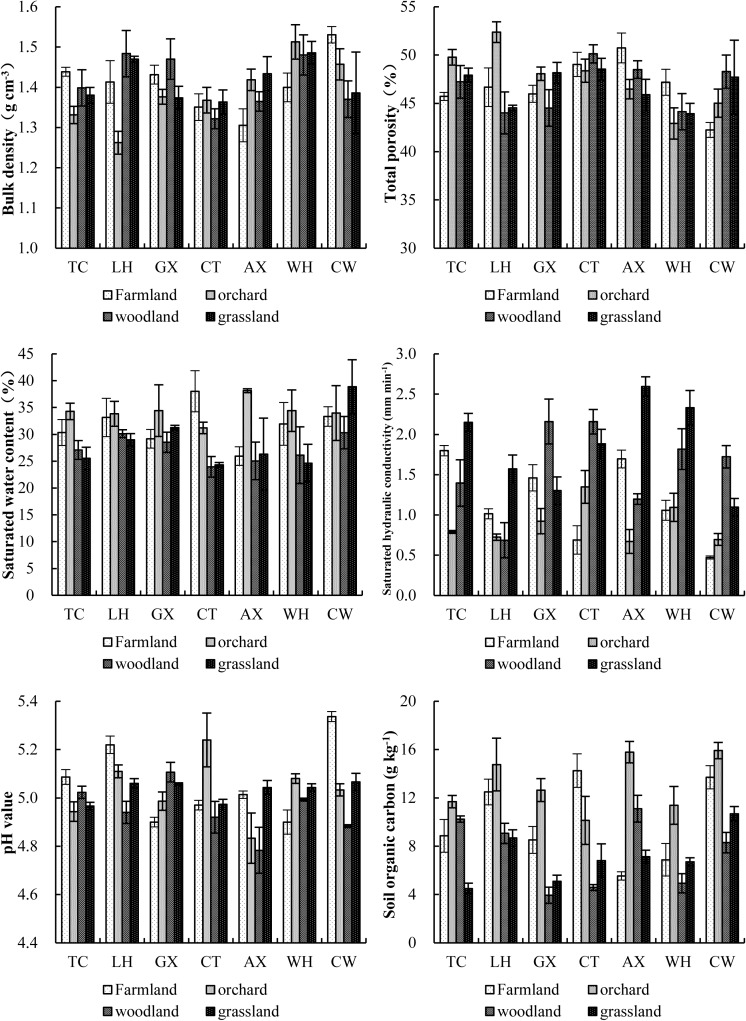
Soil physical and chemical properties of different land-use patterns in the alluvial fans of collapsing gullies

Our data analysis demonstrated that soil layers had a significant effect (P<0.05) on soil physical and chemical properties (Fig 4). Specifically, the soil BD varied greatly among the four land-use patterns in the seven alluvial fans. The highest soil BD for the land-use patterns in each county was found in the farmlands in TC (1.44 g cm^-3^), woodlands in LH (1.48 g cm^-3^), woodlands in GX (1.47 g cm^-3^), orchards in CT (1.37 g cm^-3^), grasslands in AX (1.43 g cm^-3^), orchards in WH (1.51 g cm^-3^) and farmlands in CW (1.53 g cm^-3^), and the lowest was in the orchards in TC (1.33 g cm^-3^), orchards in LH (1.26 g cm^-3^), grasslands in GX (1.37 g cm^-3^), woodlands in CT (1.37 g cm^-3^), farmlands in AX (1.31 g cm^-3^), farmlands in WH (1.40 g cm^-3^) and woodlands in CW (1.37 g cm^-3^). However, the soil total porosity (TP) exhibited an opposite trend to the soil bulk density in the corresponding samples. Our data analysis revealed that the highest soil TP for the four land-use patterns in each of the seven regions was found in the orchards in TC (49.77%), orchards in LH (52.37%), grasslands in GX (48.16%), woodlands in CT (50.13%), farmlands in AX (50.74%), farmlands in WH (47.18%) and woodlands in CW (48.29%). Differences were found in the soil-saturated water contents of the four land-use patterns (Fig 4). The range in the soil-saturated water content was 25.53–34.28% in TC, 28.98–33.83% in LH, 28.52–34.42% in GX, 23.95–38.03% in CT, 25.0–38.14% in AX, 24.65–34.41% in WH, and 30.32–38.86% in CW. Compared with that in the other land-use patterns of alluvial fans, the saturated hydraulic conductivity was significantly higher for grasslands in TC (2.15 mm min^-1^), grasslands in LH (1.57 mm min^-1^), woodlands in GX (2.16 mm min^-1^), woodlands in CT (2.16 mm min^-1^), grasslands in AX (2.60 mm min^-1^), grasslands in WH (2.33 mm min^-1^) and woodlands in CW (1.72 mm min^-1^).

As shown in Fig 4, pH values ranged from 4.78 to 5.34, indicating that the pH values of alluvial fan soils are relatively low, possibly due to the acidic nature of the granite parent materials. For the four different land-use patterns of all the collapsing gullies, the lowest pH values were 4.97, 4.94, 4.90, 4.92, 4.78, 4.90 and 4.88 for TC, LH, GX, CT, AX, WH and CW, respectively. There were significant differences in the soil organic carbon content of the various land-use patterns in the order grasslands (4.50 g kg^-1^) < farmlands (8.86 g kg^-1^) < woodlands (10.25 g kg^-1^) < orchards (11.69 g kg^-1^) in TC; grasslands (8.68 g kg^-1^) < woodlands (9.06 g kg^-1^) < farmlands (12.49 g kg^-1^) < orchards (14.76 g kg^-1^) in LH; woodlands (3.94 g kg^-1^) < grasslands (5.10 g kg^-1^) < farmlands (8.52 g kg^-1^) < orchards (12.65 g kg^-1^) in GX; woodlands (4.58 g kg^-1^) < grasslands (6.81 g kg^-1^) < orchards (10.14 g kg^-1^) < farmlands (14.26 g kg^-1^) in CT, farmlands (5.54 g kg^-1^) < grasslands (7.12 g kg^-1^) < woodlands (11.11 g kg^-1^) < orchards (15.79 g kg^-1^) in AX; woodlands (4.94 g kg^-1^) < grasslands (6.71 g kg^-1^) < farmlands (6.88 g kg^-1^) < orchards (11.38 g kg^-1^) in WH; and woodlands (8.29 g kg^-1^) < grasslands (10.69 g kg^-1^) < farmlands (13.71 g kg^-1^) < orchards (15.92 g kg^-1^) in CW.

### Relationship between fractal dimension and soil particle-size distribution

**Fig 5:**
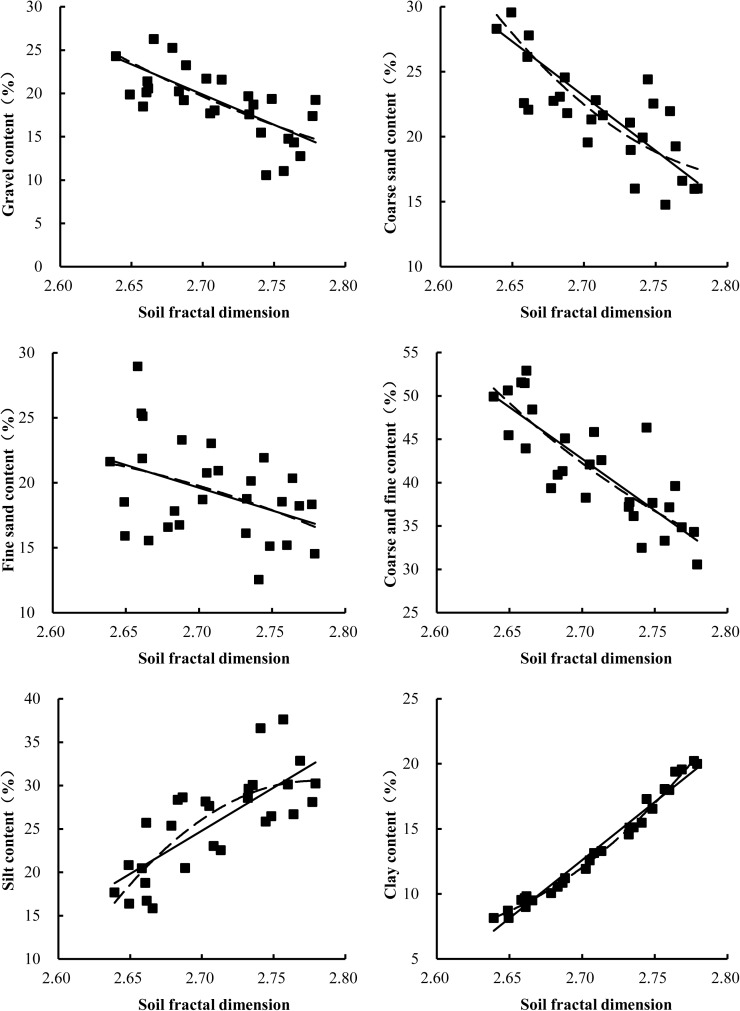
Correlations between soil fractal dimension and soil particle-size distribution.

Linear and nonlinear regression analyses were performed to determine the strength of the relationships between D values and the contents of gravel, coarse sand, fine sand, silt and clay as well as the relationships between D values and general soil characteristics in the soil of alluvial fans of collapsing gullies (Fig 5). The linear regression analysis showed that the fractal dimension of PSD had a strong positive correlation with the clay content (R^2^ = 0.976, P<0.001), a weak positive correlation with the silt content (R^2^ = 0.578, P<0.01), a remarkable negative correlation with the content of gravel (R^2^ = 0.494, P<0.01) and the total content of coarse sand and fine sand (R^2^ = 0.685, P<0.01), and a weak negative correlation with the contents of coarse sand (R^2^ = 0.623, P<0.01) and fine sand (R^2^ = 0.171, P<0.01). Additionally, as shown in Table 3, all the correlation coefficients of the linear regression equation were lower than those of the second-degree polynomial regression equation, especially the correlation coefficient (0.578) of the linear regression equation and the correlation coefficient (0.615) of the second-degree polynomial regression equation based on the silt content.

**Table 3:** Regression and correlation analysis of the soil fractal dimension with soil physico-chemical properties.

	Regression equations	R^2^	Regression equations	R^2^
Gravel content	*y* = −69.688*x* + 208.02	0.494	*y* = 126.46*x*^2^ − 755.07*x* + 1136.4	0.496
Coarse sand content	*y* = −84.092*x* + 250.17	0.623	*y* = 362.18*x*^2^ − 2047*x* + 2909.2	0.637
Fine sand content	*y* = −34.987*x* + 114.09	0.171	*y* = −82.379*x*^2^ + 411.49*x* − 490.71	0.172
Coarse and fine content	*y* = −119.08*x* + 364.26	0.685	*y* = 279.8*x*^2^ − 1635.5*x* + 2418.4	0.690
Silt content	*y* = 99.421*x* − 243.65	0.578	*y* = −725.15*x*^2^ + 4029.6*x* − 5567.4	0.615
Clay content	*y* = 89.345*x* − 228.63	0.976	*y* = 318.89*x*^2^ − 1639*x* + 2112.6	0.991
Soil organic carbon	*y* = 71.735*x* − 184.89	0.777	*y* = 284.01*x*^2^ − 1467.5*x* + 1900.2	0.792
pH value	*y* = 0.6844*x* + 3.1643	0.063	*y* = 16.939*x*^2^ − 91.12*x* + 127.52	0.110
Bulk density	*y* = 0.033*x* + 1.3169	0.001	*y* = −1.1636*x*^2^ + 6.3394*x* − 7.2258	0.001
Saturated water content	*y* = 79.352*x* − 184.49	0.639	*y* = 11.141*x*^2^ + 18.968*x* − 102.69	0.639
Total porosity	*y* = −1.246*x* + 50.307	0.001	*y* = 43.909*x*^2^ − 239.22*x* + 372.67	0.001
Saturated hydraulic conductivity	*y* = −11.775*x* + 33.274	0.788	*y* = 8.9556*x*^2^ − 60.312*x* + 99.022	0.789

### Relationship between fractal dimension and soil characteristics

**Fig 6:**
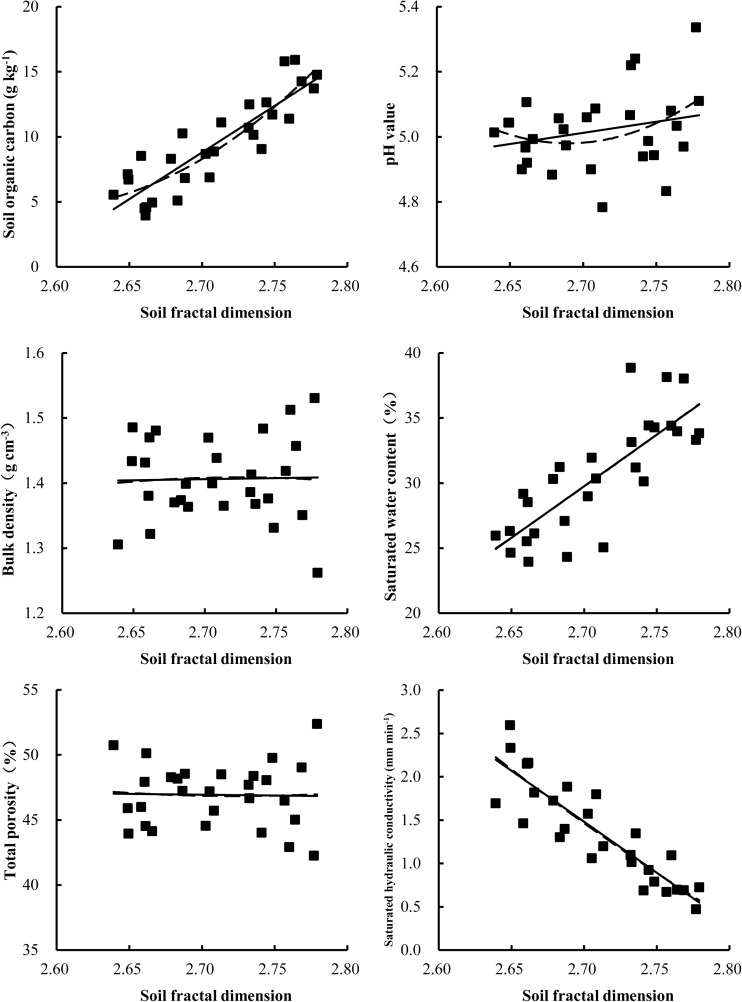
Correlations between soil fractal dimension and soil physical and chemical properties.

The occurrence of collapsing gullies severely affected the physical and chemical properties of alluvial fan soils. The linear regression results in Fig 6 clearly show that the fractal dimension of PSD had a significant positive correlation with the SOC (R^2^ = 0.777, P<0.001) and saturated water content (R^2^ = 0.639, P<0.01) and a weak positive correlation with pH (R^2^ = 0.064, P<0.05) but a strong negative correlation with saturated hydraulic conductivity (R^2^ = 0.788, P<0.001). However, the results revealed no significant correlation with bulk density and total porosity. Accordingly, an association of D values with the physico-chemical properties of the studied soil was established, indicating that the lower the D value, the worse the physico-chemical properties of the soil will be. Therefore, the characteristics of D reveal a physical degradation process of soils in the expansion of land desertification and soil nutrient reduction in the alluvial fans of collapsing gullies.

## Discussion

Soil PSD, a key determiner of soil particles and pore characteristics (such as size, number, and geometry), is commonly used in soil classification and the estimation of various related soil properties due to its relationship to soil water movement, structure, productivity, and erosion [10, 39, 60]. Thus, identifying changes in soil PSD may provide useful information for further research regarding soil protection, soil recovery mechanisms, and other soil science topics of alluvial fans of collapsing gullies in hilly granitic regions. Fractal geometry is increasingly applied as an effective tool to describe soil structure, dynamics, and physical processes, facilitating a better understanding of the performance of a soil system [7, 61–62]. The current study demonstrates that the fractal dimension of PSD increases as soil becomes finer in texture [23, 63–65].

### Soil particle-size distribution and fractal dimension

In this study, land-use pattern was found to produce highly significant effects on the fractal dimensions of PSD, indicating that it is a primary factor influencing the PSD of alluvial fans in collapsing gullies. Collapsing gullies develop quickly and suddenly erupt, depositing a large amount of gravel and sand in alluvial fans [37–38, 40, 46]. Most of these deposits are composed of quartz, feldspar and mica, exhibiting signs of hard weathering [66]. Land-use patterns in alluvial fans significantly affected the extent of water loss and soil erosion, resulting in variations in PSD and other soil properties. In the present study, soil PSD varied greatly among the four land-use patterns of the seven alluvial fans of collapsing gullies. Compared with other land-use patterns, orchards and farmlands generally had higher concentrations of fine particles (silt and clay), but their gravel contents were lower, whereas the gravel content in grasslands was greater than 20% for the four land-use patterns, except in AX and CW. These results indicate that agricultural land use is an effective means for soil recovery in alluvial fans, possibly because land-use patterns, especially orchards and farmlands in this study, can accelerate the weathering of minerals such as feldspar [40], leading to a reduction in the concentration of coarse particulate matter in the soil and an increase in the concentration of fine particles. In addition, different fertilization measures and vegetative litter contribute to the formation of humus in orchards and farmlands during the planting process, which increases the percentage of clay particles and changes the soil particle distribution.

The diversity of PSD due to different land-use patterns and positions in alluvial fans results in differentiated fractal dimensions. In this study, the D values are relatively low compared to those reported in several previous studies [10, 39], which is in agreement with the report by Cheng et al. (2007) [67], who found that the fractal dimension of the soil originating from granite was lower than that of the soil from other parent materials. However, higher soil D values from certain land-use patterns, especially farmlands and orchards, correspond well to improved soil conditions. Previous studies indicated that natural grasslands, woodlands and shrublands typically had higher fractal dimensions of PSD than farmlands in the surface soil profile. The high D values of these land-use types can be attributed to greater vegetation cover [32, 62] and reduced human activities [68], which decreases soil erosion and increases the soil clay content. However, in this study, most of the woodlands and grasslands had lower fractal dimensions than farmlands (Table 2), possibly due to the large quantity of gravel and sand in the sediments of alluvial fans, which are resistant to erosion. Thus, grasslands are not an effective land-use type for soil restoration. However, it can also be inferred from the low soil fractal dimensions that the sand concentrations of alluvial fan soils increase after gullies collapse. Agricultural land can minimize soil erosion and retain fine soil particles, indicating that reasonable planning is important for the recovery of soil productivity in alluvial fans. In addition, we assessed the determination coefficient of the linear fitting for the fractal model (Eq 2) when it was applied to the different land-use patterns in the alluvial fans. As shown in Table 2, there is a strong linear trend in the data for each of the land-use patterns and the presence of strong correlations as indicated by R^2^ values greater than 0.90 in all cases. These results are similar to those of Liu *et al*. (2009) [62] and Xiao *et al*. (2014) [33], suggesting the effectiveness of using the fractal dimension of PSD as a descriptor for soils.

### General soil characteristics of alluvial fans

Land-use patterns were also found to have significant effects on the soil physico-chemical properties of alluvial fans in collapsing gullies. Soil bulk density (BD), an important physical property of soil, directly affects soil porosity, pore size distribution, soil water, and fertilizer gas thermal change [69]. In this study, no significant consistency was found for the four land-use patterns. Soil BD was highest for farmlands in TC and CW but lowest in AX and WH, whereas soil total porosity (TP), an indicator of the response of soil structure [70], exhibited a contrasting trend at the corresponding locations. A higher soil TP implies better soil structure in the alluvial fans; thus, we can conclude that the soil structure of orchards is better in TC, LC and GX. Soil saturated water content is the maximum amount of water a soil can hold against gravity. Increasing the soil saturated water content is an important method to combat drought in granite soil [71]. The soils of orchards had the highest saturated water content except in CT and CW, suggesting that orchards increased the water holding capacity in alluvial fans. However, the saturated water content of woodlands and grasslands was relatively lower, suggesting the soils in these areas can easily become arid and require frequent irrigation. Saturated hydraulic conductivity is a challenging soil hydraulic property to describe because it can change by many orders of magnitude over short distances [72]. Contrary to the general trend of saturated water content, the saturated hydraulic conductivity of grasslands and woodlands is relatively higher. This difference may be due to the high content of gravel and sand in the soils of these land-use patterns, which leads to increased soil macropore size, facilitating the generation of preferential flow and an increase in Ks with an increased water penetration rate in the soil. The organic carbon content was significantly higher in farmlands and orchards than in woodlands and grasslands. As a key attribute of soil fertility and productivity, the generally high levels of organic carbon can primarily be attributed to the massive return of crop residues to agricultural fields and the increased application of organic fertilizer. Therefore, agricultural land may contain more organic carbon, which is consistent with the study by Deng et al. (2015) [40].

### Relationship of fractal dimension with soil particle-size distribution and soil characteristics

Numerous studies have demonstrated that the fractal dimension of PSD increases as the soil becomes finer in texture (i.e., from sand to loam to clay) [32–33, 64–65]. In this study, the results of the linear regression equation showed that D values had a significant and positive correlation with soil clay and silt content and a remarkable negative correlation with soil gravel, coarse sand and fine sand. These results illustrate that a higher clay or silt content in an alluvial fan would lead to a higher fractal dimension, whereas a higher sand content would lead to a lower fractal dimension. These observations are consistent with a previous study by Song et al. (2015) [64]. In the process of a collapsing gully, soil clay often accounted for the largest proportion of material loss. Decreased clay concentration is an indication of severe soil erosion, and the fractal dimension of soil PSD decreases with a decrease in clay concentration, indicating that the soil fractal dimension can reflect soil erosion to a certain extent and can be used as an indicator of soil erosion, including collapsing gully events. Additionally, land-use patterns can effectively demonstrate the use value of soils in alluvial fans. In the present study, with an increase in fine soil particles (clay and silt) in farmlands and orchards, the fractal dimension increased, whereas the soil fractal dimension in woodlands and grasslands remained low, indicating that agricultural land is more conducive to the formation of fine particles.

Based on the results of a linear regression equation and second-degree polynomial regression equation, D values had no correlation with bulk density and porosity, which is similar to the findings of Xia et al. (2015) [39]. Several previous reports state that D reflects the spatial filling capacity of soil based on the distribution of soil particles [21–22]. In other words, the smaller the soil particle diameter is, the greater the spatial filling capacity of the soil will be, which corresponds to higher fractal dimension values of PSD. However, the bulk density and porosity are also affected by other factors in addition to the soil particle size distribution in the present study. D values exhibited a significant positive correlation with saturated moisture content, which implies that the greater the D value is, the stronger the water holding capacity will be in the soil of the alluvial fan. Additionally, there was a significant negative correlation between D and the soil saturated hydraulic conductivity. This finding implies that the greater the concentration of fine soil particles, the slower the water infiltration will be, which is beneficial to soil and water conservation.

Soil grain size can reflect the ability to maintain plant nutrition. The chemical composition of fine particles involves many plant nutrient elements, and some of them can be released slowly into the plant. A significant positive correlation was observed between soil organic carbon and D values, implying that finer soil particles promote the binding of soil organic matter [73]. Soil organic carbon content is one of the most important indexes in soil quality assessment and soil structure stability [74–75]. In this study, the correlation between D and SOC indicates that the D values can be used to assess changes in the soil quality and soil structure of alluvial fans with different land-use patterns.

In addition, as shown in Fig 4, all the R^2^ values of the second-degree polynomial regression equation were higher than those of the linear regression equation, indicating that the second-degree polynomial equation is more adequate for describing the correlations between soil fractal dimensions and soil particle size distribution. Our results also indicate that D can be used to characterize the uniformity of soil texture among different land-use patterns.

## Conclusions

Based on our analyses of PSD, D and the relationships of D values with soil physico-chemical properties for different land-use patterns in the alluvial fans of collapsing gullies, the following conclusions are summarized:

1. The four land-use patterns in the alluvial fans studied exhibit a significant effect on soil PSD and physico-chemical properties. Compared to grasslands and woodlands, farmlands and orchards generally contain more fine soil particles (silt and clay) and fewer coarse particles.
2. The four land-use patterns vary significantly in the fractal dimension of soil PSD. The soil fractal dimension is lower in grasslands but higher in orchards relative to the other two land-use patterns. The average soil fractal dimension of grasslands is 0.08 units less than that of orchards. Additionally, farmlands and orchards are lower in bulk density but higher in porosity, whereas woodlands and grasslands are lower in saturated moisture content. All four land-use patterns have high saturated hydraulic conductivity.
3. Fractal dimension exhibits significant linear relationships with the silt, clay and sand contents and soil properties; it also shows positive correlations with the contents of clay (R^2^ = 0.976, P<0.001), silt (R^2^ = 0.578, P<0.01), organic carbon (R^2^ = 0.777, P<0.001) and saturated water (R^2^ = 0.639, P<0.01) but negative correlations with the contents of gravel (R^2^ = 0.494, P<0.01) and coarse sand (R^2^ = 0.623, P<0.01) as well as saturated hydraulic conductivity (R^2^ = 0.788, P<0.001). However, fractal dimension has no significant correlation with pH, bulk density and total porosity. Thus, higher contents of silt and clay and a lower content of sand result in higher fractal dimension values.

The results of this study demonstrate that fractal dimension analysis of soil particle-size distribution is a useful approach for the quantitative evaluation of different land-use patterns in the alluvial fans of collapsing gullies in southern China. The correlation of fractal dimension with soil structure (bulk density and total porosity), soil moisture (saturated water content and saturated hydraulic conductivity) and soil nutrients (pH and organic carbon) provides a novel method for quantifying aggregation and the effects of land use on soil quality.

## Supporting information

S1 Table Soil physical and chemical properties of different land-use patterns in the alluvial fans of collapsing gullies. (XLSX)
